# Habit Formation in Wearable Activity Tracker Use Among Older Adults: Qualitative Study

**DOI:** 10.2196/22488

**Published:** 2021-01-19

**Authors:** Wei Peng, Lin Li, Anastasia Kononova, Shelia Cotten, Kendra Kamp, Marie Bowen

**Affiliations:** 1 College of Communication Arts and Sciences Michigan State University East Lansing, MI United States; 2 Clemson University Clemson, SC United States; 3 University of Washington Seattle, WA United States; 4 State of Michigan Lansing, MI United States

**Keywords:** habits, action planning, coping planning, activity trackers, fitness trackers, continued use, mobile phone, older adults, health behavior, mHealth

## Abstract

**Background:**

Wearable activity trackers are popular devices used to motivate behavior change. Wearable activity trackers are especially beneficial for encouraging light physical activity such as walking, which is an ideal behavior for older adults or individuals who cannot be physically active at moderate and vigorous levels. A common problem is that people do not continue to use these wearable devices, with initial behavioral change gains eroding as people disengage. Limited research is available regarding the continued use of wearable activity trackers. The habit formation literature may provide insights into the long-term use of wearables and other health informatics devices.

**Objective:**

This study aims to uncover the mechanism underlying the long-term continued use of wearable devices among older adults through the theoretical lens of habit formation.

**Methods:**

In-depth interviews were conducted with 20 participants who were aged 65 years or older and had used wearable activity trackers for more than 6 months to understand their experiences and the strategies they employed to support continued use.

**Results:**

Thematic analysis of data revealed 8 themes related to habit formation, including aspects in initiation and goal setting, use of contextual cues, action planning, and coping planning. Long-term users tended to have meaningful initiation of wearable activity trackers. They usually started with a small behavioral change goal and gradually increased it. They used consistent time and locational cues to make the use of wearable activity trackers routine. Long-term users also used creative contextual cues and reminders to facilitate action planning, engaged in coping planning to deal with anticipated problems, and had a positive mindset and inventive strategies for managing unfulfillment and lapses.

**Conclusions:**

The results of this qualitative study of long-term users of wearable activity trackers suggest specific ways to enhance long-term habit formation among older adults. These best practices by long-term users can inform the future design of technology-based behavior interventions.

## Introduction

### Long-term Use of Health-Related Devices

A multitude of devices and gadgets, from the early pedometer to the wearable activity tracker, have been developed over the past few decades to help individuals change from a sedentary existence to a more active and healthier lifestyle. Wearable activity trackers are sensor-enabled devices used for monitoring physical activity, sleep, and other fitness and health-related states to facilitate behavioral change and improve health [1]. Examples of wearable activity trackers include commercial products such as Fitbit, Garmin, or Apple Watch. Studies have shown that using these devices can be effective in motivating and facilitating physical activity increases [2-6]. Wearable activity trackers have the potential to facilitate and sustain behavior change to help individuals be more physically active [5-9]. If individuals have continued engagement with wearable activity trackers, it is more likely that these devices can positively affect behavior change.

However, the problem is that many people start using the devices but abandon them quickly. For instance, wearable activity trackers have not lived up to expectations, with most people discontinuing their use within 6 months of starting [10-12]. Most of the empirical research focuses on identifying reasons why people did *not* use or continue to use personal informatics devices, such as forgetting to put it on, inconsistency between users’ anticipations and the real functionalities of the device, mismatch between devices and the user’s self-concept, difficulties in managing the device over time, reaching saturation in knowledge from the monitoring, or achieving behavior change goals and thus losing the sense of benefit from wearable activity trackers [13-19].

Limited research is available regarding continued use of these devices [20-23]. Without the continued use of wearable activity trackers, their benefits cannot be fully achieved. Therefore, understanding why individuals continue to use wearable activity trackers and what strategies they employ to do so is critical for wearable activity trackers to reach their full potential for health promotion.

### Habit Formation and Maintenance

The habit formation literature provides insights into the long-term use of wearable activity trackers and other health informatics devices [24-27]. Habit is defined as “behavioral patterns enacted automatically in response to a situation in which the behavior has been performed repeatedly and consistently in the past [28],” following what is commonly accepted in the field of psychology. Once a habit is formed, individuals engage in action *automatically* without requiring much cognitive effort [29]. People do not need to *remember* to engage in the action; they do so out of *second nature* [30]. A meta-analysis revealed a moderate to strong effect size (random effect *r*=0.46) for the correlation between the behaviors of healthy eating and physical activity and the self-reported habit index [31]. A 12-week randomized control trial found that habit formation was significantly related to taking more steps per day and adherence to the intervention [27]. The critical issue is to understand the process of habit formation and maintenance. Therefore, the aim of this study is to investigate *how* long-term users of wearable activity trackers develop the habit of using wearable activity trackers to manage their physical activity.

Habits are acquired through the repetition of behavior in a consistent context [32,33]. The habit formation process involves 3 progressing phases: initiation, learning, and stability. In the initiation phase, decisions are made to perform a behavior and the context in which it will be performed is selected. Instilling sufficient motivation, setting small and realistic goals, and choosing a simple action that will help individuals move toward the goal are essential in this phase [34]. Before actions are repeated in a consistent context, intention to act, action planning, and coping planning are needed to prepare the initiation of habit formation. Action planning involves detailed specification of the features of situations and intended responses to the situation. One example of action planning is the *if-then* rule; if situation X happens, then one will do Y [35]. Action planning helps to establish a link between contextual cues and behaviors. Reminders can help people remember their plans [36]. Another type of planning, coping planning, deals with anticipating difficulties that might hinder the action and creating concrete plans to overcome potential obstacles [37].

The second phase is the learning phase in which the behavior is repeated to strengthen the context-behavior association. The critical factor for habit formation is the consistent context cue: repetition must occur in association with the same event triggers, similar time and location, or similar people or objects. For instance, people usually form a habit of closing their garage door when they drive out of the garage. Through numerous repetitions of pushing the button to close the garage door at the same event of driving out of the garage (ie, a contextual cue), people do not need to think of pushing the button deliberately. Sometimes, people cannot remember whether they have closed the garage door or not and just return to find out that the garage door is indeed closed—they did it out of habit automatically.

When a new action is performed, a mental association between the situation (contextual cues) and the action is created. Through repetition, the association of contextual cues and behaviors strengthens; dependence on conscious attention or motivational process to perform an action is reduced, and only the contextual cues are sufficient for the action. Behavior achievement promotes continued repetition [28].

The third phase of habit formation is the stability phase in which the behavior persists over time with minimal effort and deliberation [34]. At this stage, the habit has formed, and its strength has peaked and plateaued. Once the habit is formed, even after conscious motivation or interest fades away, the action or behavior may still be performed when contextual cues are present. The habit concept yields potential for health-related behavior change, which is typically a long-term process characterized by the initiation of new behavior and maintenance (ie, repetition) over time [28].

In this study, we focus on the first 2 phases, as initiation and learning phases bring most insights to individuals whose habit has not formed. We propose to study how long-term *power users* developed the habit of using a wearable activity tracker regularly to manage physical activity via the following research questions (RQ):

- RQ1: How did long-term users initiate the use of wearable activity trackers?
- RQ2: How did long-term users set goals in the context of tracker use?
- RQ3: What consistent contextual cues did long-term users use to form the habit of wearing wearable activity trackers?
- RQ4: How did long-term users engage in action planning and coping planning to support the continued use of wearable activity trackers?

## Methods

### Overview

We adopted a qualitative approach in this study because limited evidence exists to examine the habit formation process and specific strategies for continued use, particularly for older adults. Our qualitative approach with long-term users has the benefit of gaining an in-depth understanding of the habit of continued technology use and behavior maintenance. This study was approved by the institutional review board of Michigan State University (IRB# x16-1583eD).

### Participants

This study is situated in the context of wearable activity tracker use among older adults. In-depth interviews were conducted with 20 older adults (aged 65 years and above) who had used wearable activity trackers for more than 6 months. Older adults were selected for this study for several reasons. First, previous research has found that wearable activity trackers are beneficial for enhancing physical activity among older adults [3,38,39]. Wearable activity trackers typically track step counts, and walking is a significant contributor to physical activity in older adults [40]. Increasing physical activity in older adults can help stave off chronic disease development and progression. The very act of wearing a wearable activity tracker that monitors and encourages movement may remind older adults to be more proactive in increasing their physical activity. This study focused on long-term *power users* or individuals who have used behavioral modification technology over 6 months. Previous research on technology-based behavioral interventions primarily investigated efficacy and barriers while missing the opportunity to identify successful strategies employed by *power users*. These best practices by long-term users can inform the future design of technology-based behavior interventions.

Long-term wearable activity tracker users were recruited in January 2017 by distributing a survey to a Qualtrics panel of older adults (aged 65 years and above) who had an experience with wearable activity trackers. A total of 314 respondents completed the survey, of which 163 indicated that they had used wearable activity trackers for more than 6 months. Among the 163 long-term *power users*, 71 stated that they were interested in our follow-up interview study exploring their experiences with wearable activity trackers. Of the 71 participants, 20 were randomly chosen for the in-depth phone interview, which took place from February to April 2017. Among the 20 participants, the average age was 67.95 years (SD 2.01) and 55% (11/20) were female. The average length of the wearable activity tracker used in the sample was 31.9 months (SD 25.64), with 15 reporting that they used a Fitbit. Other devices reported by the participants included Garmin, Miband, and a cell phone. Detailed demographic information of the interviewees is listed in Table 1.

**Table 1:** Participant demographic information (n=20).

Participant ID	Gender	Age (years)	Race or ethnicity	State
1	Female	67	White	Minnesota
2	Female	66	White	Oregon
3	Male	67	Asian	Hawaii
4	Female	68	White	Michigan
5	Male	65	White	Pennsylvania
6	Male	66	White	Alabama
7	Female	68	White	Arkansas
8	Male	70	White	North Carolina
9	Male	68	White	New Jersey
10	Female	67	White	Ohio
11	Female	71	White	Colorado
12	Female	70	White	Georgia
13	Male	66	Hispanic or Latino	Texas
14	Female	69	White	California
15	Female	72	White	Tennessee
16	Female	66	White	Texas
17	Male	69	White	Indiana
18	Male	65	White	Maryland
19	Female	70	White	Wisconsin
20	Male	68	White	Nevada

### Procedure

The 20 randomly selected participants were contacted via telephone by a researcher to confirm the availability and length of the wearable activity tracker use. We sent an informed consent document a week in advance and then read an abbreviated version over the phone and obtained verbal consent. At the end of the first interview, the participants were asked to use their smartphones to take photographs that provided insights into the use of wearable activity trackers in their lives. As the participants were long-term users of wearable activity trackers and their habit of use had already developed, these photos helped the participants recall and become more conscious of certain automatic behaviors for the interview discussion. The participants then texted or emailed the photos with annotation to the research team. The photo-taking and sharing exercise lasted for a week, and then a follow-up interview was conducted. After completing the first interview, participants received a US $30 Target, Walmart, or Amazon gift card via mail or email. After completing the photo-taking exercise and the second interview, the participants received a US $50 gift card to the store of their choice. This study reports the elements in the interviews that pertain to habit formation for the continued use of wearable activity trackers.

### Interview Materials

Semistructured interviews were conducted. The interview guide was developed based on the habit formation literature [32-34,41]. For the first interview, researchers began with the consent process. Once individuals agreed to participate, researchers started with ice-breaker questions. Then the participants were asked questions related to general use, habit formation, and data usage of the wearable activity tracker. Example questions are included in Table 2. At the end of the interview, researchers explained that they would participate in a photo-taking task for about a week to capture what, where, when, and why they used their trackers and strategies for long-term use of the trackers. The second interview included questions about the photos taken by each participant, the context of tracker use, benefits of wearable activity trackers, and successful strategies of long-term use. Table 2 summarizes the interview guide topics and provides examples of questions.

**Table 2:** Interview guide and example questions.

Interview			Example questions
**First interview**			
	**Interview topics**		
		Icebreaker	Can you describe how you use your tracker on a typical day?
		General use	What features do you use the most and least and why? Did you set a goal for yourself when using the wearable activity tracker? How do you feel when you don’t reach your goals?
		Habit formation	Tell me the story of when you started using your tracker. What made you decide to start using one? Why have you continued to use your tracker for so long? What got you in the habit of using your tracker? Are there times of the day you use or notice your activity tracker more than other times of the day? What makes it difficult to use the activity tracker on those days? How do you deal with the difficulties? If a friend wanted to use an activity tracker every day for a year, what are the three main pieces of advice you would tell your friend?
		Tracker data usage	How often do you check your tracker data? Do you share your tracker data? How do you use the data provided by the tracker?
**Second interview**			
	**Interview topics**		
		Photo diary opening question	What physical activity were you doing when you took this photo?
		Context	Where were you when you took this photo?
		Benefits of wearable activity trackers	How did you feel physically while doing this activity? How do you see the wearable activity tracker contributed to your physical health? Were you with anyone when you used the activity trackers? How do you see the wearable activity track contributed to your social life?
		Successful strategies of long-term use	Please explain to us the successful strategies you employed to continue using the wearable activity tracker, which are captured in these photos.

### Data Analysis

Both rounds of interviews were recorded and transcribed verbatim with the use of a web-based transcription service, Rev. The transcribed interview data were systematically approached by applying the principles of thematic analysis [42-45]. Thematic analysis is a flexible, efficient, and accessible method that is widely used in qualitative psychology to generate rich data that provide social and psychological interpretations of phenomena and illuminate unpredicted insights relevant to the topic of the study [42]. While acknowledging the subjective nature of collected data, we used a postpositivist empirical approach to data analysis, which implies that the reality can be known through the accounts of humans—in our case, experiences and voices of long-term users of wearable activity trackers [42,43]. This approach is described as essentialist and experiential [42,43]. Furthermore, we used a mix of inductive and deductive techniques in data coding and theme generation. Data coding was predominantly an inductive, data-driven process to identify a wide array of patterns in participants’ descriptions of long-term tracker use. Aggregating the codes within each theme was approached both inductively and deductively; we applied the literature of habit formation (deduction) and accounted for unanticipated findings (induction) to define themes [42,43].

Thematic analysis of the transcripts was performed in 5 stages, as suggested by previous literature [42,43]. These stages included (1) becoming familiar with the transcribed interview data, (2) coding, (3) outlining initial themes, (4) reviewing and revising themes, and (5) defining final themes [42,43]. First, each author read the transcripts to become familiar with the data (stage 1). Then, the transcripts were iteratively analyzed by 4 researchers using NVivo 10 and NVivo 11 software (QSR International) for qualitative data analysis [46,47]. All 4 researchers analyzed the same 3 randomly selected transcripts to generate nodes (stage 2). The researchers were then divided into 2 groups, and each group analyzed half of the transcripts. After completing the initial coding, the researchers reviewed the nodes and discussed disagreements and discrepancies. If 2 researchers coding the same transcript could not come to an agreement, a third researcher was invited to resolve the disagreement. The nodes were iteratively compared and revised during the coding process. Throughout the discussions of nodes, the initial theme formation started (stage 3). Themes were then generated, reviewed, and finalized based on the final nodes identified, and most relevant quotes were selected in support of the themes (stages 4 and 5). As the scope of the interviews was broader than in this study, only nodes and themes relevant to the RQs in this study are reported.

Trustworthiness and consistency of the findings were established through a number of procedures. The instrument for the interview was developed and refined by all members of the research team and reflected the goals of this study. The interviewers were properly trained to recruit participants and conduct telephonic interviews. As the data collection continued, the researchers met weekly to discuss the study progress and the highlights of the interviews. A codebook was established to analyze the data and derive nodes. Multiple coders coded the transcripts and met regularly to discuss progress and resolve inconsistencies and disagreements. Finally, quote relevance was carefully assessed to support the final themes [45].

## Results

### Overview

A total of 8 themes were identified to answer the 4 RQs. Table 3 summarizes the themes with exemplary quotes from the participants.

**Table 3:** Summary of identified themes.

RQ^a^	Themes	Example quotes
RQ1: How did long-term users initiate the use of wearable activity trackers?	Meaningful initial start	“She [The physician] laid it out to me there, end of February: ‘You’re going to have a choice. We’re going to give you injections, or it’s borderline [diabetes]. Exercise and eating right can reduce it.” (Participant 20)
RQ2: How did long-term users set goals?	Goal setting: start with a small goal and gradually increase	“The tracker came preset with the 10,000 step goal. I think that’s what the American Heart Association recommends. I didn’t really push my steps for that goal because if I was walking, I would still have some pain in my chest……After I had that stress test after a year is when I really started picking up the pace and doing a lot more and working on the 10,000 steps every single day. There have been days where it’s tracked 15,000 so I would be significantly over the goal some days. It did take me a while to work up to it.” (Participant 4)
RQ3: What consistent contextual cues did long-term users use to form the habit of wearing wearable activity trackers?	Consistent cues: use time and locational cues to make it a routine	“If you develop a routine and a habit by having a place you put it every night, an agreement with yourself that it’s the first thing you do when your feet hit the ground in the morning is put it on, then it will become a habit. Anything you do for 15 days in a row becomes a habit. If you can do it for 15 days, you probably will continue to do it.” (Participant 15)
RQ4: How did long-term users engage in action planning and coping planning to support the continued use of wearable activity trackers?	Action planning: creative contextual cues Action planning: use reminders Coping planning: anticipate problems and have a plan to deal with it Coping planning: mindset for managing unfulfillment and lapses Coping planning: try to have fun and try something new	“While we were talking, I’ve been walking some because I knew I still lacked a few steps. And so, that’s right. Anytime I talk on the phone, you know, since we all have the cell phones, I walk while I talk.” (Participant 9) “When you set up the account you can turn on the alerts and the alerts can go to your phone and can go to also your email. That is... I did both and it’s a duplicate because the email goes to the phone, too, but...whatever gets my attention at the moment, that’s fine. And then I go and charge. And it doesn’t take long to charge.” (Participant 10) “I carry one with me. I have it in my bag that I carry to work, but I do have two chargers. So that’s probably a good idea, too, to have more than one. I know one person I know has it in their car. Charges it in their car.” (Participant 7) “Put it on and don’t be discouraged the first few days or even weeks or months you don’t reach what you want, because it’s like everything else. It’s something new. It’s something you’ve never done before.” (Participant 1) “Maybe in the spring so you can hear the birds and just do something like that to motivate you and look at the other side of town. When you walk over and say, hey I haven’t been down in this area for quite a while. You've got to come up with something that motivates you to walk or to see something different, or whatever.” (Participant 19)

^a^RQ: research questions.

### Meaningful Initial Start

Some long-term users have been physically active for a long time, and wearable activity trackers are just another tool for them to monitor their physical activity automatically. However, many of our long-term users became physically active after they started to use wearable activity trackers. Most of the participants indicated that they took it very seriously when they started using the wearable activity tracker to increase their physical activity levels. Although some purchased the wearable activity tracker on their own and some received it as a gift, the majority of participants experienced significant events in their life that made them realize that they must do something to change their lifestyles. The primary event was declining health. Some participants experienced a gradual decline, followed by a significant health event such as surgery. Some participants were given either severe warning or gentle encouragement from their health providers to exercise to address the health issue:

Anyhow, but so what I’m saying is over those years my health had been declining without me really recognizing it because it wasn’t just an abrupt thing. With the heart surgery, that being an abrupt event in my life, I realized that I definitely needed to do more to try to get the exercise and the sleep, everything that I needed as well as diet.

Participant 4

She [The physician] laid it out to me there, end of February: ‘You’re going to have a choice. We’re going to give you injections, or it’s borderline [diabetes]. Exercise and eating right can reduce it.

Participant 20

The aforementioned quotations were from individuals facing severe health challenges. Although not all participants had such dramatic life-threatening events that triggered them to begin using wearable activity trackers to help them exercise more, they mentioned other health-related reasons such as weight loss or weight management, better diet, increased physical activity and metabolism, and general preventative practice for healthy retirement life. It seems that for individuals to start forming a habit of long-term use, they needed to see a meaningful purpose behind the action to make up their mind and be determined to make a change.

### Goal Setting: Start With a Small Goal and Gradually Increase

Goal setting was one of the most mentioned technological features of wearable activity trackers that assisted users in becoming long-term users. Before a habit is formed, goal setting is important. Different wearable devices on the market have a goal-setting feature and the goal is typically set at 10,000 steps for everyone by default. However, this goal target of 10,000 steps may not work for everyone, especially older adults or individuals who are relatively sedentary to start. Our participants did not always follow the default goal suggested by the device and instead set realistic goals based on their conditions and gradually modified their goals. Each round of goal setting and goal accomplishment increased participants’ confidence and self-efficacy in using wearable activity trackers to monitor physical activity. It likely helped them form a habit and become long-term users. Participants acknowledged the importance of starting with a reasonable step count. Health problems often limited participants; however, gradually increasing their goal enabled the participants to meet their goals:

She [My daughter] has set it 10,000, which I found was just a little bit too much for me, so I changed it down to 4,000... Well, get it down to where it’s more reasonable to start out with, and then move up... I would say set your goals realistically. Don’t set them because you think this is what you should do.

Participant 1

The tracker came preset with the 10,000 step goal. I think that’s what the American Heart Association recommends. I didn’t really push my steps for that goal because if I was walking, I would still have some pain in my chest...After I had that stress test after a year is when I really started picking up the pace and doing a lot more and working on the 10,000 steps every single day. There have been days where it’s tracked 15,000 so I would be significantly over the goal some days. It did take me a while to work up to it.

Participant 4

### Consistent Cues: Use Time and Locational Cues to Make It a Routine

It sounds effortless: “just put it on.” However, many individuals stop using wearable activity trackers because they cannot remember to put it on [19]. What are the tricks among the long-term wearable activity tracker users? As the habit formation literature predicts, volitional goal setting is the first step before the autonomous habit is formed, and consistent cues that trigger behaviors repeatedly move individuals toward habituation. Commonly discussed temporal cues are morning and night routines. Long-term users typically put on the wearable activity trackers the first thing in the morning. A time cue of going to bed reminds some users to plug in the wearable activity tracker to charge so that it is available for use the next day:

If you develop a routine and a habit by having a place you put it every night, an agreement with yourself that it’s the first thing you do when your feet hit the ground in the morning is put it on, then it will become a habit. Anything you do for 15 days in a row becomes a habit. If you can do it for 15 days, you probably will continue to do it.

Participant 15

Another essential contextual cue is the location. Participants leave the wearable activity trackers at the same location at night or for charging, usually a place where they can easily spot, for example, nightstand, besides the clothes. Time and locational cues enabled participants to develop the habit of wearing a wearable activity tracker. Consistent cues regarding time and location provided the older adults a framework on which to develop a habit. As many of the participants had previously worn watches, the process of developing a routine for wearing a wearable activity tracker was identified as similar to wearing a watch. Many of the participants recognized that once they were able to develop the habit of wearing the wearable activity tracker, it became an automatic process:

It’s just right there on the, where I lay my clothes out, so it’s like okay, this is part of it. It’s like putting your earrings in. This is just what you do.

Participant 9

You don’t take it off except to charge it or take a bath or shower; you put it in a spot where you’re gonna put it right back on if you do have to take it off. Just do it all the time and it becomes a habit.

Participant 7

I would say that the most important thing is the issue of getting into a regimen. They can get on an automatic thing like I do. Basically in the morning you just put it on. You don’t even think about. It’s like putting on my watch. It’s an automatic thing. That’s number one.

Participant 18

### Action Planning: Creative Contextual Cues

Action planning helps establish the link between contextual cues and behaviors. Action planning involves detailed specification of the features of situations and intended responses to the situation, creating an if-then rule. Because long-term users have planned ahead of time what to follow once the contextual cues are triggered, they could easily engage in the planned actions. The long-term users in the study had a number of creative contextual cues, such as commercial breaks, phone conversations, and ice and snow outside. For instance, if there was a commercial break or if participants were having a phone conversation, they would get up and walk. Wearable activity trackers most likely did not help participants plan action or create *if-then* rules; however, the devices provided the concrete behavior consequence (eg, step count), which is a way to validate their action planning:

At night or when I start watching TV in the evening, I get up during the commercials. All the commercials I walk.

Participant 12

While we were talking, I’ve been walking some because I knew I still lacked a few steps. And so, that’s right. Anytime I talk on the phone, you know, since we all have the cell phones, I walk while I talk.

Participant 9

In the winter, because of the ice and snow, I don’t get as much activity, steps and stuff like that, but I will walk around the house to try and get to my, the number of steps. If somebody would drive by and look in the window, they’d see me back and forth, back and forth, like I’m pacing.

Participant 17

### Action Planning: Use Reminders

Long-term users also acknowledged that it was easy to forget to put on the wearable activity trackers when they first started, especially after they took it off for charging. One of the strategies they adopted was using reminders, for example, on their phones. Some participants used the alert feature available from the wearable activity tracker website to alert their phone or email regarding the need to charge the tracker. Participants tried to avoid charging the tracker last minute as often they would forget to put on the tracker. They actively planned to charge the tracker while engaging in sedentary behaviors and use reminders:

When you set up the account you can turn on the alerts and the alerts can go to your phone and can go to also your email. That is... I did both and it’s a duplicate because the email goes to the phone, too, but...whatever gets my attention at the moment, that’s fine. And then I go and charge. And it doesn’t take long to charge.

Participant 10

### Coping Planning: Anticipate Problems and Have a Plan to Deal With It

Coping planning, anticipating difficulties, and developing plans to overcome challenges was also a common practice among long-term users. They anticipated potential obstacles that might prevent them from wearing the wearable activity tracker and planned for how to deal with them. Once wearing wearable activity trackers becomes a daily routine, or in the participants’ words, “becoming automatic,” the long-term wearable activity tracker users are challenged to maintain a certain level or even increase physical activity levels. One participant identified coping planning to address the potential of a dead battery:

I carry one with me. I have it in my bag that I carry to work, but I do have two chargers. So that’s probably a good idea, too, to have more than one. I know one person I know has it in their car. Charges it in their car.

Participant 7

### Coping Planning: Mindset for Managing Unfulfillment and Lapses

Adopting a new behavior does not always mean that the behavior will be continuously maintained. Throughout behavior change, maintenance, and dealing with occasional lapses are essential [48]. Sometimes, people can be discouraged when lapses happen, and they stop using wearable activity trackers altogether [15]. The long-term tracker users shared that they were prepared for occasional unfulfillment and lapses with a positive mindset. The expectation of ups and downs helped prevent users from quitting at the beginning of forming the habit:

Put it on and don’t be discouraged the first few days or even weeks or months you don’t reach what you want, because it’s like everything else. It’s something new. It’s something you’ve never done before.

Participant 1

Besides the positive mindset to view early road bumps as inevitable and prepare for it, long-term users also tried to find a positive angle to view lapses. For instance, they focused on the larger picture, such as a weekly goal achievement, rather than on a 1-day relapse:

I think just consistency is the most important thing. Wear it every day and there might be some days when you can’t achieve your goal but if you look at it on a weekly basis, and say ‘Okay, my goal for the week is 70,000 steps, every day it’s 10,000. If something happens, if you’re sick one day or you have business there or whatever and you only get in the 5,000, if you get in 12,000 two other days or 12,500, then you’re still at your goal for the week.’ That’s one thing I kept in mind is that you look at the goal on a weekly basis as well as on a daily basis.

Participant 4

### Coping Planning: Try to Have Fun and Try Something New

Engaging in the same behavior over and over again can be dull, even after one has already established as a habit. Most of our long-term users wear wearable activity trackers to track walking steps as a way to monitor their activity level. Taking the same route for walking every day certainly is a good way of keeping the number of step counts; however, it also can make wearing the wearable activity tracker unnecessary as today’s step count would not be much different from yesterday’s. One reason for the abandonment of a wearable activity tracker is that it is no longer needed once users have gauged their routine activity level [15]. The participants tried to explore new routes or new environments, making physical activity more interesting and justifying the need to wear the wearable activity tracker to gauge step counts:

Maybe in the spring so you can hear the birds and just do something like that to motivate you and look at the other side of town. When you walk over and say, hey I haven’t been down in this area for quite a while. You've got to come up with something that motivates you to walk or to see something different, or whatever.

Participant 19

## Discussion

### Summary of Findings

This qualitative study obtained valuable insights from 20 long-term wearable activity tracker users who were at least 65 years old to inform future development of interventions to increase wearable activity tracker use and physical activity among older adults. The value of this study is in its focus on factors that contribute to the long-term use of wearable activity trackers among older adults, explored through the theoretical framework of habit formation. Numerous studies have focused on the initial stages of health-related technology use [19,49,50], often neglecting the issue of long-term, sustainable engagement. Existing studies examining the continued use of wearable activity trackers and other personal informatics devices primarily focus on reasons for abandonment rather than long-term use strategies [21-23]. Studying sustainable wearable activity tracker use in the older adult population is seen as especially beneficial, as this particular group may greatly benefit from physical activity [40,51]. Older adults are susceptible to developing chronic diseases such as diabetes, cardiovascular diseases, and obesity, which could be protected against by regular physical activity [52]. Factors contributing to long-term use, in particular, successful strategies to support habit formation and long-term use, have implications not only for wearable activity tracker use but also for other information technology facilitated behavior change.

Our qualitative results confirmed 4 types of strategies, consistent with the habit formation literature, which were used by our participants to become long-term users of wearable activity trackers. Specifically, they set up realistic and adaptive goals in the early phase of habit formation and creatively relied on contextual cues to trigger goal-related behaviors automatically. They engaged in action planning, a form of mental stimulation for how to act when contextual cues are triggered, translating intentions, and goals to action. Finally, they deliberately did coping planning, another type of mental stimulation to anticipate obstacles and alternative behaviors to overcome those obstacles.

### Goal Setting

Among the features that are available in wearable activity trackers, goal setting was one of the most mentioned helpful features. Goal setting, as an important step in the early phase of habit formation, is common in most wearable activity trackers. The default goal of 10,000 steps is not always obtainable for older adults, especially those who are struggling with health issues. One of the suggestions to help form sustained wearable activity tracker use habits that came from the interviewees was to start with realistic goals. If one wants to make a change, the desired outcome is achievable [34]. This approach not only encourages older adults to move but also boosts their self-efficacy as they achieve their daily activity goals [28,34]. Previous research on health apps has also shown that the ability to set customizable goals helps boost individuality, which contributes to the sustained motivation of improving physical fitness or weight loss [53]. For older adults who prioritize positive emotions and emotionally meaningful goals in life, adaptive goals that are attainable at different stages of behavior change may be especially important for their sense of meaning and well-being [54]. Designers of personal health informatics devices may consider realistic and adaptive goal setting based on users’ initial use data rather than providing a default goal for everyone. For instance, when users start using the wearable activity tracker, it is crucial to analyze their age, current activity level, and health status to craft a realistic goal and then adaptively modify the goal based on the daily step count data from the user.

### Consistent Contextual Cues

Automaticity emerged as the core *element* of the habit formation process. Most of the tricks to maintain use of wearable activity trackers revolved around making wearable activity tracker use routines less effortful or noticeable. Repetition and consistency play an important role [26]. To get into the habit, one must put the wearable activity tracker on at the same time every day. Participants tended to keep their wearable activity trackers in the same place, put them on as part of a grooming routine, or charge them at the same time. These consistent contextual cues helped them establish a routine of wearing wearable activity trackers that were incorporated into their everyday lives. Specifically, these time and location cues are the bases for action planning as they signal *when* and *where* actions should take place. Most recently, the HeartSteps study found that contextually tailored activity suggestions can be helpful for users to increase their step counts [55]. By using an Android-based mobile health app to send users actionable activity prompts tailored for their current context, including the time of day, day of the week (weekday or weekend day), location (home, work, and other), and weather (suitable for walking outside or not), delivered at common times when individuals can participate in physical activity, such as during commute, midafternoon, or after dinner, researchers found that delivering activity walking suggestions (vs providing no suggestions) increased the subsequent 30 min step count by 14% to 24% on average. In light of these findings, delivering activity suggestions on wearable activity trackers may have a more substantial effect on physical activity, considering that individuals have more immediate and constant access to them. The HeartSteps study also found that the effects of contextually tailored activity suggestions decreased as the study duration increased. Nevertheless, the study was a 6-week intervention, which may not be enough time to keep users engaged and form the habits of participating in physical activity.

Most existing wearable activity trackers do not have built-in features that facilitate the establishment of consistent contextual cues for habit formation. Long-term users in our study established these contextual cues based on their routines. Designers of future wearable activity trackers or personal health informatics devices may consider adding in features to facilitate users to establish contextual cues to enable habit formation and later use an automatic process. For instance, GPS is available in many wearable activity trackers, and smart wearable activity trackers can use location data to infer location cues. If wearable activity trackers send push notifications to invite users to annotate these contextual cues, future push notifications can be sent as reminders when the contextual cues are triggered. In addition, contextual cues are very much individualized. In other words, each individual may have different *when* and *where* cues that are meaningful for them. Wearable activity trackers may offer a list of cues to action for users to identify personally feasible cues.

### Action Planning

Action planning uses the so-called *if-then* rules to establish the link between contexts and behaviors. It includes specific situational parameters (*when*, *where*) and a sequence of actions (*how*). When such links are repeated over time, behaviors might be elicited automatically when relevant situational cues are encountered. Our participants engaged in action planning to use creative contextual cues and reminders for prolonged use of wearable activity trackers, adding empirical evidence for the theory of the habit formation process [28]. There is already evidence showing the effectiveness of action planning for behavior change in multiple domains, including physical activity [37,56]. Action planning should be explored further as a strategy for the continued use of wearable activity trackers or other health informatics devices.

Action planning was done mainly by long-term users without any assistance from their wearable activity trackers, which is a gap where designers of future wearable activity trackers or health informatics devices can fill. The strategies used by those long-term users may be programmed into wearable activity trackers to facilitate habituation and continued use. For instance, wearable activity trackers may send push notifications suggesting planned behavioral enactment if the *when* and *where* contextual cues are triggered. For instance, one example of action planning among our participants was to get up and walk some steps when television commercials were on. A television commercial is a *when* contextual cue that triggers the *if-then* rule for walking behavior. Future wearable activity trackers may prompt the users to choose a few from a list of *if-then* rules that are personally relevant to them for action planning. As evidenced by the HeartSteps study, along with similar just-in-time interventions delivered at critical decision points that are most suitable for facilitating individuals’ health behavior change [57], there is great potential for integrating action planning with contextual cues in initiating and maintaining the process of habit formation as we move toward the future of precision health [58].

Next-generation wearable activity trackers or health informatics may even be equipped with smart sensors to detect the *when* and *where* contextual cues and provide just-in-time information to nudge the users to engage in the planned behavior. For instance, a smartwatch equipped with sound sensors can detect television sounds and send just-in-time prompts that it is time to walk when the commercial is on. It should be noted here that these suggested action planning scenarios are not about wearing wearable activity trackers but about increasing physical activity. Nevertheless, the primary goal of any wearable activity tracker is to increase physical activity, and the discussed scenario cannot be implemented if the user is not wearing a wearable activity tracker.

### Coping Planning

Coping planning is a mental simulation of overcoming anticipated barriers to action. Good intentions or well-set goals are more likely to be translated into action when people develop success scenarios and preparatory strategies for approaching difficult tasks. A systematic review shows that coping planning is an effective technique for promoting health-related behavior change, especially in combination with action planning [59]. Relapse and barriers to action are always expected. The key is how to deal with them when they happen. Our participants stressed that disruptions in wearable activity tracker use should not be a reason to stop using wearable activity trackers. They noted the importance of continuing to use wearable activity trackers even if step levels were lower than desired or individuals had forgotten to use the wearable activity tracker for a while. This insight suggests that behavioral disruptions are to be expected; however, *getting back on the horse* and resuming wearable activity tracker use are integral to continued long-term wearable activity tracker use. This finding is also consistent with previous evidence that occasional deviation of behavior does not seriously impair habit formation as long as additional repetition continues [41].

Nevertheless, coping planning before disruption or relapse is essential for habit formation. Coping planning ensures the continuance of the habit formation process and the effectiveness of other elements, including goal setting, environmental cues, and action planning, for habit formation. The lack of premeditations or deliberate mental efforts inherent in the coping planning process may constitute a critical reason that people fail to stick with the usage of wearable activity trackers. Nevertheless, coping planning is also the most often neglected component among studies on the habitual use of wearable activity trackers [27,60]. Considering its significance in helping participants bridge the gap between their intentions and actions to engage in health behaviors [59], lack of support for coping planning is a weak spot of existing wearable activity trackers and health informatics devices. Designers may consider incorporating coping planning, especially coupling coping planning with different contexts that can be sensed by wearable activity trackers (eg, lack of data for several days) or contextual data (eg, weather information) to better facilitate habituation. For instance, when the weather forecast indicates snow for the next 5 days and the GPS of the wearable activity tracker shows that the user is mostly walking outside, a push notification may be programmed to send to the user to plan for indoor walking before snowfall.

It is sometimes difficult for individuals to anticipate barriers and devise coping planning ahead of time. Another approach might be to monitor behaviors and obstacles and plan for coping strategies when the same obstacles occur in the future. For example, a wearable activity tracker might detect that the user did not wear a wearable activity tracker for several days or did not log the targeted steps for several days. The wearable activity tracker could then send a push notification to the user to annotate why this happened and how to deal with this if this happens again in the future.

### Limitations

Although the results of our study provide suggestions for enhancing long-term wearable activity tracker use habit formation, our study is not without limitations. First, we interviewed 20 older adult long-term wearable activity tracker users from across the United States to discern how they formed long-term wearable activity tracker use habits. Although the participants were geographically dispersed across the United States, they were skewed to *younger* older adults, with an average age of approximately 68 years. Older adults in their 80s and beyond are likely to be different from those in their upper 60s in terms of wearable activity tracker use and long-term wearable activity tracker use. In addition, 18 out of 20 participants of our sample were White. We know little about how the patterns identified in these interviews might differ among a more diverse racial and ethnic sample. Future research is needed that follows a diverse sample of older adults over time as they begin to use wearable activity trackers to determine how habits are formed in wearable activity tracker use. Second, our study relied on self-report, which may be subject to recall bias given that the average time of wearable activity tracker use for our long-term wearable activity tracker users was almost 3 years, although we implemented a photo diary to facilitate recall, especially recall of habitual behaviors. Third, we relied on habit formation literature to examine the continued use of wearable activity trackers. However, there are other models available that predict continued use of technology, such as the expectation-confirmation model [61], which may be adopted in future research regarding the continued use of wearable activity trackers and other health informatics devices. Fourth, the focus of this study is on the continued use of wearable activity trackers, not necessarily habitual physical activity. Nevertheless, some participants equated wearing wearable activity trackers with physical activity, and some participants noted that wearing a wearable activity tracker was the first step in behavior change. We deliberately only included interview results about wearing wearable activity trackers; however, wearing wearable activity trackers and engaging in physical activities were intertwined in some interviews. Fifth, further advancement of wearable technology in the population of older adults may uncover additional barriers to continuous technology use. For example, advanced wearable activity trackers may require older adults to share personal information with the technology owners, which may create privacy-related concerns. Sixth, although the participation incentive was US $80 in total, it matched with the level of time commitment and effort because the participants needed to engage in 2 interviews that lasted for 60 to 90 min each and they also needed to keep a photo diary of wearable activity tracker use. The possibility that the financial incentives would introduce a potential bias toward keeping up the appearance of long-term users is low because only long-term users identified by the Qualtrics survey received recruitment information for the interview study. In other words, participants disclosed their length of wearable activity tracker use in the Qualtrics survey before they were aware of the interview study and its incentives. Finally, as this study focuses mainly on the behavioral factors that are conducive for forming habits of using wearable activity trackers, we did not consider the personality and motivational aspects of habit formation. Research has found that self-control, self-determination [62-64], conscientiousness, and a growth mindset [65] are predictive of healthy habit formation, which, in turn, is predictive of physical activity or exercise behaviors. For example, the positive relationship between trait self-control and physical activity is mediated through behavioral automaticity, a key component of habit [62]. The positive relationships between self-control and positive life outcomes, such as healthy eating and consistent sleep, are also mediated through beneficial habits [66]. Another study found that self-determined motivations are more predictive of behavioral automaticity than nonself-determined motivations across 12 common daily behaviors [63]. Even though we did not directly study physical activity, it is arguable that these personality and motivational factors may also have similar effects on the habit of wearing wearable activity trackers, which is a necessary tool for facilitating behavior change. We suggest that these relationships should be explored in more detail in future research.

### Conclusions

The results of this study shed light on a range of factors that may contribute to long-term wearable activity tracker use among older adults. Although contextual factors, such as where the wearable activity tracker is placed and how often it is charged, and technological factors, such as build-in goals and reminders, are important. Participants also noted the importance of self-initiated actions and coping planning for forming wearable activity tracker use habits. The results of this study provide practical recommendations for behavior change interventions. In summary, the results of this qualitative study of long-term *power users* of wearable activity trackers suggest specific ways to enhance long-term habit formation for wearable activity tracker use among older adults. The findings add rich qualitative insights into the emerging literature on habit formation. Strategies identified by long-term *power users* could be useful in the design of next-generation wearable activity trackers or technological devices to facilitate behavior change.

## Abbreviations

RQ: research question
